# Book Reviews

**Published:** 1907-04

**Authors:** 


### BOOK REVIEWS.

PRACTICAL DERMATOLOGY, a condensed manual of diseases of the skin, designed for the use of students and practitioners of medicine, by Bernard Wolff, M.D., Clinical Professor of Diseases of Skin in the Atlanta College of Physicians and Surgeons; Editor of the Atlanta Journal-Record of Medicine, Ex-President of the Atlanta (Fulton County) Society of Medicine; Ex-Secretary of the Georgia State Commission on Tuberculosis, etc.

The author's long experience as a teacher and his reputation as a dermatologis are sufficient evidence that the contents are well-chosen, and the essentials of dermatology are adopted in this small volume as far as the subject is of interest to the ordinary student and practitioner and is filled with important points of information, culled from the best authors and judiciously adapted to practical needs.

The work is eminently practical and while its purpose is not intended, nor can it be regarded as a treatise on dermatology, it covers a field of greatest importance, dealing fully and concisely as it does with that class of dermatoses for which the physician is most often consulted, and met with in general practice, a class of disease which is exceedingly common and annoying to many, but which is given too little attention by the average practitioner.

From the standpoint of treatment it covers the field most thoroughly; the uses of electricity, especially galvanism and the static modalities and high frequency currents, also the use of the X-ray radio and photo-therapy, in its relation and to treatment of diseases of the skin is clearly and thoroughly handled—the choice of method—its dangers, advantages and disadvantages, are clearly and practically considered—the directions and indications for general local treatment are clear, and that advised in the text is supplemented by appending to each chapter a number of prescriptions, which have proven of especial value in the hands of the author, while the whole of the third section of the book is devoted to Formulary culled from the works of various authors, giving completeness and breadth of view to the subject, making it in all a work of the greatest interest, value and help to the student and busy practitioner. L. C. R.

---

AN EPITOME OF DISEASES OF THE NOSE AND THROAT. By J. B. Ferguson, M.D., of the New York Post-Graduate Medical College Hospital; Laureate of the Royal Academy of $1.00, net. Lea Brothers & Co., Publishers, Philadelphia and New York, 1907. (Lea's Series of Medical Epitomes. Edited by Victor C. Pedersen, M.D., New York.)

The author has presented in concise and practical form the diagnosis and treatment of diseases of the throat and nose. He has planned the book to be helpful to the under-graduate and post-graduate medical student in gaining familiarity with laryngological work, and likewise to the general practitioner, who is often called upon to treat diseases of this region, and who needs to have the chief points in diagnosis and treatment concisely placed at his command. All these classes of readers will appreciate the systematic arrangement, the clear directions for examination, the illustrations of preferable instruments and of diseases, and the abundant formulæ for the best medication.

---

A TEXT-BOOK OF THE PRACTICE OF MEDICINE. For Students and Practitioners. By Hobart Amory Hare, M.D., B.Sc., Professor of Therapeutics and Materia Medica in the Jefferson Medical College of Philadelphia; Physician to the Jefferson Medical College Hospital; Laureate of the Roayl Academy of Medicine in Belgium and of the Medical Society of London. Author of A Text-Book of Practical Therapeutics; A Text-Book of Practical Diagnosis, etc. In one very handsome octavo volume of 1120 pages, 131 engravings and 11 full-page plates in colors and monochrome. Second edition, revised and enlarged. Cloth, $5.00, net; leather, $6.00, net; half morocco, $6.50, net. Lea Brothers & Co., Philadelphia and New York, 1907.

One of the most uncommon and highly valuable mental faculties is judgment of relative importance, an ability to conceive ideas distinctly enough to measure them, and to picture them in their proper perspective. Such a faculty collects, arranges, sorts, drops what is unimportant, and seizes what is essential. Such qualities embodied in a book account for keen public appreciation, and this is the explanation of the fact that two very large printings and now a thoroughly revised edition of Hare's Practice have been demanded in less than two years. The author has written for medical students of all ages. He knows the needs of the undergraduate and the best mode of presenting a subject to his mind by reason of the fact that he has been teaching clinical medicine and therapeutics for nearly a quarter of a century, and the same length of active hospital and private practice gives the weight of experience which attracts the physician in need of counsel. Well-proportioned consideration is given to the theory and principles of medicine as underlying, explaining and leading up to the main objective point, namely, the practical application of the knowledge. Accordingly, particular pains have been taken to present methods of treatment clearly, and in such a way that they may be put directly into practice. Such have been the characteristics of the work from its original issue. In this new edition every line has been revised, anything already rendered obsolete by the rapid march of medicine has been eliminated, and all trustworthy advances have been incorporated, so that the volume, as it stands, is representative of its subject to date. Of its authority and resourcefulness, nothing need be said.

---

DISEASES OF THE LUNGS. Designed to be a practical presentation of the subject for the use of students and practitioners of medicine. By Robt. H. Babcock, A.M., M.D., author of "Diseases of the Heart and Arterial System;" until recently Professor of Clinical Medicine and Diseases of the Chest, College Physicians & Surgeons, Chicago, etc. Twelve colored plates and 104 text illustrations. Publishers: D. Appleton & Co., New York.

Diseases of the lungs are very important on account of their frequency and because the simpler ones, when neglected, may become serious and even incurable before a correct diagnosis has been made. The obscurity of certain of these affections require knowledge of the pathology, dexterity in making physical examinations and a judicial, discriminating mind to determine their character, the prognosis and the treatment indicated.

The excellence of Dr. Babcock's work enables us to commend it to the favorable consideration of our readers without reserve. Although comprehensive, it is practical and covers the entire field in a most satisfactory manner and is free from unnecessary, tiresome academic discussions. The drawings and cuts are very good and really illustrate the text. By careful studying this book we believe every physician can obtain more satisfactory results from this class of diseases and we are sure there will be more pleasure in treating them.

---

### A WISH.

"I wish," said Tripp,
Who had the grip,
"I wish to Gawd I knowed
Some way to close
My dog-gone nose,
Or make the thing stay blowed."

---

The Craggs research prize of £50 (about $250) will be awarded in October, 1907, to a past or present student of the London School of Tropical Medicine, who, during the year, October, 1906, to October, 1907, has made the most valuable contribution to tropical medicine. The fact of the work having already been published will not disqualify. Contributions, which must be written in English, must be in the hands of the medical superintendent of the school, Dr. C. W. Daniels, on or before October 1st.

